# Editorial: Non-pharmacologic approaches to inflammation in the critically ill

**DOI:** 10.3389/fmed.2026.1822282

**Published:** 2026-03-30

**Authors:** Ronaldo C. Go, Themba Nyirenda

**Affiliations:** 1 Rutgers Robert Wood School of Medicine, New Brunswick, NJ, United States; 2 Section of Critical Care and Internal Medicine, Robert Wood Johnson University Hospital in Hamilton, Hamilton, NJ, United States; 3 Hackensack School of Medicine, Nutley, NJ, United States

**Keywords:** critically ill, delirium, electrical therapy, inflammation, light therapy, non-pharmaceutic interventions, resuscitation, shock

A lesson learned from the COVID-19 pandemic is that critically ill patients utilize the most resource out of all healthcare groups, and require efficient resource allocation (1). When resources are limited, providers sought to maximize benefit, minimizing harm and unfair disadvantages, while juggling moral concern, reciprocity, and instrumental value (2). The burden of the disease was far greater in developing countries than in high-income countries, middle-aged and older adults and those with limited access to adequate healthcare (3). Pharmacologic treatment was extrapolated from established protocols from other diseases, but that included their costs and adverse effects. The high utilization of the critically ill can linger post-hospitalization in a chronic inflammatory state (4, 5).

A common sequalae of inflammation is delirium, which has been associated with prolonged hospitalization, and increased mortality compared to patients without delirium (6). It is a result of disruption of various pathways associated with normal cognitive function, which may be a result of direct neuroinflammation or indirectly through the decreased blood flow or neurotransmitter dysregulation associated with inflammation. Due to the inconsistent outcomes from pharmacologic treatment of delirium, there has been a shift toward prevention and early detection (Chan and Corso) (7, 8). Chan and Corso performed a systemic review of 26 studies (*n* = 8,831) examined the prevention and treatment of delirium and found equivalent efficacy between pharmacologic and non-pharmacologic methods. These non-pharmacologic interventions include promotion of regular sleep wake cycles, Minimizing invasive sensory stimulation, promotion of regular reorientation and adequate pain management (Chan and Corso) (8).

The interest in non-pharmacologic approaches to delirium is still evolving. A study by Zheng et al. is a bibliometric analysis of light therapy as an intervention for delirium. Two hundred and twenty papers were reviewed, and the United States showed the most publications, with John Hopkins as the most productive institution, and *Nursing in Critical Care* had the highest number of publications (Zheng et al.).

Another type of non-pharmacologic intervention is the direct use of electricity. Electrical Impedance Tomography (EIT) is a non-invasive imaging technique that reconstructs cross-section images of the body's tissues based on electrical conductivity (9). A case report details a 23-year-old male patient with obesity and a complex medical history, who presented with severe pneumonia and acute respiratory distress. Despite conventional treatment, the patient faced challenges in weaning from mechanical ventilation (Liu Y. et al.). By utilizing EIT, clinicians were able to monitor and adjust the patient's positioning and ventilation strategy, leading to optimized lung ventilation distribution. Specifically, EIT-guided prone positioning played a pivotal role in improving the patient's ventilatory mechanics and enabling successful weaning from the ventilator on day 26 of hospitalization (Liu Y. et al.). The findings indicate that EIT can significantly enhance the management of ventilatory support in obese patients, offering a personalized approach to improve respiratory outcomes (Liu Y. et al.). Most hospitals do not have EIT and resource intensive. Further research is warranted prior to widespread use.

Compared to pharmacological agents, non-pharmacologic interventions have taken a supportive role in treating inflammation. Minimizing the delivery of a pharmacologic *per se* is another type of non-pharmacologic approach to inflammation. Fluid resuscitation, via crystalloid or albumin, is an essential treatment in hypotension or shock associated with inflammation. The Sepsis guidelines 2021 recommended targeting at least 30 mL/kg of IV crystalloid fluid be given with the first 3 h of resuscitation (10). This recommendation's association with improved mortality is not necessarily universal. The FEAST study, a randomized control trial on children admitted for infection-related shock in sub-Saharan Africa showed that fluid resuscitation was associated with high 48-h mortality (11). Therefore, continued investigation into resuscitation strategies is needed. Liu S. et al. conducted a bibliometric based study that examines the status of fluid management in sepsis patients based on the Web of Science with data from 1978 to 2024. This is an area of increasing interest in recent years, with guidelines for fluid management in sepsis patients being reported to have the highest references and burst in 2016, as the analysis reports (Liu S. et al.). The United States leads the number of publications, and very few are from the sub-Saharan Africa (Liu S. et al.).

The predisposition to hypotension and response to treatment may have a genetic basis. Wang et al. conducted a genome-wide-association study using two-sample Mendelian randomization. Hypotension was identified as an independent hazard variable for delirium [*p* = 0.010, odds ratio [OR] [95% confidence interval (CI)] = 1.302 [1.066–1.592]] (Wang et al.). The presence of horizontal pleiotropy was found to have minimal impact on establishing causal relationship (*p* = 0.999), and there was no evidence to suggest heterogeneity between genetic variations (*p* = 0.379). Additionally, the leave-one-out method demonstrated the stability and robustness of this association. The presence of horizontal pleiotropy was found to have minimal impact on establishing causal relationship (*p* = 0.999), and there was no evidence to suggest heterogeneity between genetic variations (*p* = 0.379) (Wang et al.). Further studies of fluid resuscitation that are inclusive of a wider range of demographics are still needed.

Albumin is an alternative to crystalloid resuscitation. Ren et al.'s retrospective study sought to determine optimal cut-off albumin level for albumin infusion. Their study suggested that the albumin level on day 7 (HR, 0.920; 95% CI, 0.847–0.999; *p* = 0.046) and the maximum albumin level within the first 14 days (HR, 0.900; 95% CI, 0.838–0.967; *p* = 0.004) were independent protective factors for the 28-day prognosis in septic patients. Moreover, ROC curve analysis indicated that optimal target level for first 14-day maximum and on day 7 were 33.45 g/L and 27.85 g/L, respectively (Ren et al.).

Currently, non-pharmacologic interventions have predominantly taken a supportive role in attenuating inflammation in the critically ill. Response to treatment maybe intrinsic to the patient's constitution, race/ethnicity, diet, religion, and geographic location. Further studies are needed with a larger demographic inclusivity, particularly in areas where resources are limited.
